# The Dependence of Online Gambling Businesses on High-Spending Customers: Quantification and Implications

**DOI:** 10.1007/s10899-024-10329-z

**Published:** 2024-06-24

**Authors:** David Forrest, Ian G. McHale

**Affiliations:** University of Liverpool Management School, Chatham Street, Liverpool, L69 7ZH UK

**Keywords:** Online gambling, Pareto principle, Gambling regulation, Gambling harm

## Abstract

Online gambling has grown to be a significant industry but it faces regulatory threats because of perception that it is heavily dependent on a small segment of its customers who gamble heavily and at a level carrying elevated risk of harm. Employing a large multi-operator data set from Britain, which records individual transactions by some 140,000 individuals observed over one year, we are enabled to provide more precise estimates of the degree of concentration of revenue, compared with previous studies. High dependence on a relatively small number of customers is shown though there is variation from product to product in how small the group of account-holders of potential concern is. We conclude with a discussion of prospects for the industry in light of heightened awareness of gambling harm and resulting restrictions on online gambling spending introduced or proposed by governments or regulators in several jurisdictions.

## Introduction

The first betting and casino websites appeared in 1996 and 1997, mostly hosted by jurisdictions in the Caribbean and intended to serve the American market (Williams et al., 2012a). Over the decades since, online gambling worldwide has expanded to become a moderately large industry. Estimates from data collected by H2 Gambling Capital at the end of 2022 suggested that annual net customer losses (equivalent to consumer expenditure in other industries) from online gambling in Europe had reached €38.2bn. (EGBA, 2022). Recent data for Great Britain^1^, from the Gambling Commission (2022a) and based on regulatory returns, gave a figure of £6.4bn. over twelve months for net player losses in online gambling^2^, far in excess of the £3.5bn. reported for the terrestrial market.^3^ Past issues of the Commission’s *Industry Statistics* show that this was no aberration due to disruption caused by the covid pandemic but, rather, was consistent with a secular trend for the gambling market to shift increasingly to online play.

As gambling activity globally moved online, concern was soon expressed (by, e.g., LaBrie et al., 2007, 2008, and Wood & Williams, 2007) that, because it offered greater accessibility and privacy, online play might have the potential to increase the prevalence of problem gambling and/or to aggravate the harm experienced by existing problem gamblers. Empirical assessment in this area is difficult because causation is hard to establish. High reported rates of problem gambling among online gamblers may be caused by the structural characteristics of the product but, alternatively, may simply reflect that existing problem gamblers are disproportionately likely to be attracted to online play. Philander and MacKay (2014) was a rare study which allowed for the endogeneity of the choice to gamble online. It employed instrumental variables regression. The authors concluded that it was in fact online rather than offline play which was safer, perhaps because it offered protective characteristics, for example greater ease of keeping track of spending and access to self-help tools (such as setting limits on the size of future deposits into the player’s account). But more research is needed to confirm their findings and the issue remains controversial.

What is less controversial, and generally recognised, is that, because every transaction leaves an electronic data trail, online gambling may allow researchers to draw more valid conclusions about the gambling industry, and about gambling behaviour, than was possible when gambling took place, usually anonymously, only at land venues. Gainsbury (2011) argued that this potential would be achieved only if gambling companies were to be transparent in terms of providing their data to researchers. In the event, such transparency appears to have been the exception. Industry data have seldom been shared externally. Obstacles include that the industry is competitive (making businesses wary of revealing data which may inform their rivals’ strategies) and that operators may be averse to the risk of the research casting them in unfavourable light.

Despite these obstacles, we were able to access one year of transactional data from each of some 140,000 online accounts held by residents of Great Britain. The accounts were spread across seven different operators. Collectively, according to information provided to us by the Gambling Commission, these seven accounted for 85.5% of the national online betting market and 37.5% of the more fragmented national online gaming (slots games, casino games, bingo) market. We believe this to be the largest multi-operator data set from online gambling ever made available for research.

In the present paper, the research question, to be addressed by analysis of this data set, is: how dependent is the online gambling industry on its biggest customers? Since Clotfelter and Cook (1990), authors of a scattering of papers (to be reviewed in Sect. 2 below) have concluded that gambling in general is significantly more dependent on its most active customers than is typically the case in other industries. Whether and to what extent this is true is important for the future of online gambling businesses for at least three reasons.

First, in any business environment, there is risk if revenue is dependent on a small number of buyers. For example, high spending clients may be disproportionately likely to withdraw from the market during a recession.

Second, gambling is a stigmatised product and is certainly one with a potential to cause harm to those who lose control over their degree of engagement. The gambling industry must therefore operate under an implicit social licence (Gehman et al., 2017) if it is to be permitted to do business at all. Social acceptance is likely to be weakened where it is perceived that operator profits depend heavily on high spending players because high spend represents one of the strongest correlates of gambling harm (Markham et al., 2016). Indeed, it was the assumed dependence of revenue on problematic players which led Yani-de-Soriano et al. (2012) to question even whether online gambling in particular could ever reach standards of corporate social responsibility expected in other industries.

Third, these concerns over gambling harm may lead to the imposition of regulatory controls which enforce lower spending on an operator’s most lucrative customers. For example, during 2022, the Finnish state-sanctioned monopolist operator introduced a loss limit of €2,000 in a month for online slots play while the new Inter-State Gambling Treaty in Germany provides for a monthly deposit limit of €1,000 for online sports betting, where the limit is to apply across licensed operators such that it could not be evaded by using two or more different websites (ICLG, 2022). At the time of writing, the Government in Spain is consulting on a similar all-operators binding limit (Menmuir, 2023). In Belgium a €200 weekly limit on deposits came into force though on a per-website basis and with provision for players to apply for exemption (Orme-Claye, 2022). In Great Britain, the Government has proposed applying compulsory ‘detailed’ affordability checks on customers who incur a net loss of £1,000 or more in a single day or £2,000 or more over any 90-day period (Department for Culture, Media and Sport, 2023); and, in Netherlands, mandatory affordability checks for deposits exceeding €700 in a month will be introduced in 2024, with deposits in excess of 30% of income not to be permitted (Thomas-Akoo, 2024). All such measures would pose an existential threat to regulated operators if a high proportion of their revenue would be curtailed by hard limits or if high spending players migrated to the unregulated sector (unlicensed offshore sites) in the face of affordability checks or the need to apply for exemption from loss or deposit limits. These risks would be avoided were firms’ profits mainly to be derived from moderate players rather than ‘high rollers’.

For all these reasons, and in order to inform both strategic decisions in the firms themselves and public policy, it is desirable that accurate information should be available on the extent to which profitability depends on a small number of players. Indeed, in initiating its review of whether regulation should include limits on online play, the British Government explicitly called for the question to be informed by evidence of “how are online gambling losses split across the player cohort?” (Department for Digital, Culture, Media and Sport, 2020). In Sect. 2 of the paper, we will argue that previous studies of the distribution of activity across players have yielded estimates which are either unreliable because of dependence on self-report data or else not fully informative because of conceptual issues such as what measures of customer activity should be analysed and whether the question is capable of being answered by a single metric intended to summarise the full distribution of activity across gambling accounts. In Sect. 3, we provide details about our data set and Sect. 4 will present our estimates. Finally, Sect. 5 discusses their implications for the industry.

## Methodological Lessons from Prior Literature

Previous studies on the topic differ from each other in a number of dimensions. Some rely on survey data but some employ real-world data from online account records. Some attempt to capture dependence on a small proportion of customers by estimating the Pareto ratio but some of the more recent work argues for the use of the Gini coefficient as their preferred measure of concentration. Papers calculate their preferred indices of concentration using data covering different durations, from three months up to a year. And, across the range of papers, different measures of activity are used to calculate concentration, e.g. number of bets, total amount wagered or net customer loss. The different choices made by the researchers in these dimensions may reflect what was practically possible or what their specific research objective was, but they also raise conceptual issues which we discuss in this section of the paper. In presenting a literature review, we draw on the discussion to explain our choices of methods to employ when addressing the research question.

### Survey Data Versus Account Data

When gambling business was conducted mainly at land venues, individual players’ expenditure was not monitored and data allowing analysis of their patterns of play could be obtained only by asking (a sample of) them about their behaviour. Even in online settings, researchers still sometimes choose to work with survey data because their research ultimately focuses on the association between high spend and problem gambler status. Even if available, account data, whilst including exact details of spending by individuals, would not include any unambiguous indication of problem gambler status, hence the resort to surveys which ask for both estimates of spending and completion of a problem gambling screen.

Empirical investigation of the dependence of the gambling industry began even before the emergence of an online sector and set the approach for future studies using online data. Clotfelter and Cook (1990) appear to have been the first to illustrate the concentration of revenue from a gambling product using survey data. Based on polling by the *Los Angeles Times*, they estimated that the top 20% of buyers of lottery tickets delivered 65% of game revenue. This was a lower top-20% estimate than any reported subsequently in the relevant literature, whether from survey or from account data (and lower than would be expected from the ‘Pareto principle’ that, in all kinds of data sets, 20% of units frequently account for 80% of outcomes). Perhaps this reflected that lotteries are typically found (e.g. by Mazar et al., 2020) to have a relatively low association with addictive gambling and hence relatively few heavy buyers. On the other hand, highlighting a different point of reference than the top-20% could have presented a different impression: the study also included the finding that the top 10% of players accounted for fully half of all lottery revenue. Similarly, Grönroos et al. (2022) analysed data from a large-scale national survey in Finland and estimated that half of all gambling expenditure originated with a small proportion (just 4.2%) of past-year gamblers.

Fiedler et al. (2019) employed pre-existing survey data from France, Germany and Québec. The question on spending had differed across the three surveys. For example, in France, respondents had been asked about how much they ‘usually’ spent in a gambling session (and instructed not to count winnings) whereas German participants had been set the more challenging task of estimating their net spending (i.e. stakes minus winnings) over the preceding twelve months. Results are therefore not comparable across the three data sets. But Fiedler et al. reported that their preferred measure of concentration in spending, the Gini coefficient^4^ was above 0.8 in each case, compared with what they claimed to be the norm for ‘regular’ goods (0.6). In France and Québec, the spending question was also asked for individual forms of gambling. Again measuring with the Gini coefficient, the distribution of spending was least heavily skewed towards heavy players in the case of lotteries whereas poker in France and electronic gaming machines in Québec exhibited the greatest degree of concentration. Wardle et al. (2023) followed the precedent of measuring concentration by the Gini coefficient, calculated in their case from estimates of spending on a range of gambling products provided by members of an online panel of regular race and sports bettors. The value of Gini was highest for online casinos and table games in land casinos (about 0.96 in each case).

However, the ability of any of these surveys to yield reliable measures of concentration is questionable given that there is abundant evidence that respondents appear to find recall of spending on gambling difficult. For example, 514 customers of an Australian online betting site were asked to report their outcome from the wagers they had made on that site in the prior thirty days. Heirene et al. (2022) then compared responses with account data from that website. Only 4.1% reported a figure within 10% of the true figure. Even responses to the apparently simpler question of how frequently they had bet over the thirty days yielded almost as poor recall accuracy. Given that the time frame for the questions was only the immediately preceding thirty days, one might reasonably speculate that questions asked about net spending over a full year would be unlikely to provide data which could be used with confidence in estimating measures of concentration.

Further, the use of survey data typically raises questions of whether the sample was representative. Wardle et al. (2023) sampled ‘regular’ (at least monthly) race and sports bettors from an online YouGov panel, to which members are self-recruited and credited with a modest fee for participating. Williams et al. (2012b, pp. 30–31) and Pickering and Blaszczynski (2021) raised numerous concerns about the quality of the data from online incentivised panels including their tendency to yield very much higher estimates of problem gambling prevalence than those from in-person or telephone random sampling. This suggests that concentration of spending may be over-estimated because of the sample having under-representation of more recreational, lower-spending players. In the particular case of Wardle et al. (2023), the restriction of the sample to ‘regular’ sports and race bettors may also lead to inaccuracy in the estimation of the Gini coefficients because more occasional bettors were excluded from the sample.^5^

It is clear that account data provide a more reliable source than survey data. Not only do online customer records show objective data, avoiding recall errors, but they also allow extraction of the conceptually appropriate metrics. Our interest is in the distribution of spending across customers, where spending is defined as stakes minus prizes. This can be observed directly in account data. However, the question put in survey-based studies has been ambiguous in many cases. In the data used by Grönroos et al. (2022) respondents were asked to “estimate the amount of money you spent on gambling”. The question used by Wardle et al. (2023) was somewhat similar. Wardle et al. note that their question could be interpreted as referring either to amount gambled or to net loss. They acknowledge, on the basis of prior cognitive testing of such questions, that most respondents will probably have given an estimate which refers to their gross rather than their net amount spent gambling. That no responses in the data set gave a negative estimate appears to support the answers being interpreted as amount wagered because the sample was sufficiently large that it is implausible that it included no winners over a three-month time frame.

The research question for us is how dependent firms are on a small proportion of customers. For this, player losses rather than turnover (the amount gambled) is the appropriate metric for measuring revenue to the business because it is the amount customers leave behind at the operator after a trading period. It can be directly accessed in player account data. Amount gambled is likely to be a highly imperfect proxy. LaBrie et al. (2007) ordered online sports bettors with bwin by turnover and by net loss and reported only a modest Spearman rank-order correlation coefficient (+ 0.50). One reason they gave is that a greater level of activity was associated with a lower loss per euro wagered, the same pattern observed by Nelson et al. (2021) in a successor study using much more recent data from the same platform, and by Forrest & McHale (2022) in British online betting data. This is consistent with greater expertise residing with more active bettors. But, in a follow-up analysis of data from bwin’s online casino, LaBrie et al. (2008) found that, even where the majority of games were of pure chance, rank-order correlation between turnover and losses was still only + 0.70. This could be because the average game mix differs between more and less active players (games differ in pay-out rates with more volatile games typically offering lower expected value) or might to some extent reflect incentives (such as free spins) being offered more regularly to ‘loyal’ players. In any event, our research question requires measurement of the distribution of losses across players. Given that survey data tend not to be successful in collecting loss data, comparison of findings based on surveys with our findings reported below may be problematic.

Comparisons would be more appropriately made with prior studies which are specifically on online gambling using account data. For a set of customers who had opened accounts in a particular month and subsequently played a casino game, LaBrie et al. (2008) examined the distribution of the amount staked over two years. They found a discontinuity in the distribution at the 95th centile, which identified the top-5% of players by total stakes as standing out from the rest. From the tables in the paper, it can be calculated that these top-5% accounted for 49.4% of net house win.^6^

Subsequent studies using account data also reported higher degrees of concentration of activity than would be anticipated by those holding to the Pareto principle that, in many data sets, 20% of individual units account for 80% of activity. Fiedler (2012) found that 91% of the operator take on an online poker website originated with the 10% most active customers. Tom et al. (2014) used a sample of bwin customers for whom it was possible to match account data with responses to a survey which included a screen for problem gambling. Defining the ‘vital few’ as those who together contributed 80% of operator net revenue, they estimated, for example, that this group comprised only 7% of clients in fixed-odds sports betting. A similar calculation for casino games estimated the ‘vital few’ as just 4.9% of players. However, the data set used will have included individuals who had won money over the year and it is not clear from the text how the calculations accounted for this complication.

The complication introduced by the presence of winners in the data set was addressed explicitly by Deng et al. (2021). They analysed player records from the online casino operated by the British Columbia Lottery Corporation, which offers both slots and table games. Over a one-year period, 10.6% of accounts showed a net gain to the customer. It was noted that including these customers in the calculation of the Pareto ratio (defined as the proportion of operator net revenue contributed by the top-20% of players ranked by size of loss) would yield an estimate significantly above 100%. The notion that the top-20% of players were responsible for much more than 100% of the net revenue of the casino would be uninformative and would not reveal what the spirit of the analysis was trying to unveil. The anomaly arises because low- and middle-loss players in the data set do not lose enough to cover the winnings of the ‘lucky’ account-holders. Collectively, the bottom-80% of players therefore deliver negative value to the casino. This implies that the amount of casino win from the top-20% is greater than the aggregate casino win across all players, i.e. the Pareto ratio exceeds 100%.

Deng et al. (2021) resolved this problem by recoding the outcome for winning customers as zero and went on to estimate a Pareto ratio of 91.8% when measured over twelve months. However, the loss of information in this solution might be considered problematic. Suppose the online casino business is indeed heavily dependent on ‘high rollers’. In any given year, some of these will by chance finish ahead, even substantially ahead (for example, they may have won the jackpot on a slots game). But the casino will be happy to retain them for the following year because the expected value to be extracted from them remains positive. For the longer-run prosperity of the business, the ‘high rollers’ who happen to have won this year are just as important as those who lost this year. But treating them as spending zero relegates them to customers of no consequence. Any given (high) share of revenue will then be attributed to too small a proportion of customers.

### Pareto Ratio Versus Gini Coefficient

Before progressing further, we present some definitions. The Gini coefficient is derived from the Lorenz curve. A Lorenz curve is a graphical representation of the distribution of a random variable. Typically, the random variable is a measure of wealth or income, such that the x-axis of the Lorenz curve plot represents the cumulative proportion of individuals and the y-axis is the cumulative proportion of wealth (or income).

For a discrete random variable, ${y}_{i}$, $i=1,\dots n$, let the order statistics be ${y}_{\left(1\right)}\le {y}_{\left(2\right)},\dots ,{y}_{\left(n-1\right)}\le {y}_{\left(n\right)}$. With empirical cumulative distribution function F, the Lorenz curve is given by

$$L\left({F}_{i}\right)=\frac{\frac{1}{n}{\sum }_{j=1}^{i}{y}_{i}}{{\sum }_{i=1}^{n}{y}_{i}}$$

For a uniform distribution (for example, each individual has the same level of wealth), the Lorenz curve forms an $x=y$ diagonal line (a line of perfect equality). The Gini coefficient serves as a summary measure of the asymmetry of the underlying distribution of wealth. Mathematically, it is defined as the area between the observed Lorenz curve and the diagonal, divided by the total area under the line of perfect equality. As such, the line of perfect equality has a Gini coefficient of 0, and the maximum Gini coefficient value of 1 is obtained only when the distribution is completely asymmetric such that all of the wealth is held by one individual.

Researchers often focus on one particular point on the Lorenz curve, corresponding to the percentage of wealth held by the top-20% of individuals (ordered by wealth). This percentage is often referred to as the Pareto ratio, referencing a generalisation that in many contexts a high proportion of activity is accounted for by a small proportion of individuals, for example, the proportion of industry sales accounted for by the top-20% of customers or the proportion of visits to doctors accounted for by the 20% of patients with the most frequent attendance. In gambling research, Deng et al. (2021) reported the Pareto ratio (the percentage contribution of the top-20% of customers) as their chosen measure of concentration. Tom et al. (2014) reported an alternative point on the Lorenz curve, the percentage of customers responsible for 80% of operator net revenue. Either focus reflects the influence of the rule-of-thumb, popular in marketing education, that 20% of buyers account for 80% of revenue. Either appears to hint that the share of the top-20% in the gambling sector should be compared with a benchmark of 80% which applies across ‘ordinary’ industries which sell ‘non-addictive’ goods. Whether 20–80 is truly the norm across industries is, however, questionable. Based on data from 339 American companies, McCarthy and Winer (2019) found that, on average, whether a business provided physical goods or else services, the top-20% of customers contributed 67% of turnover. Whether this would translate to a greater or lower share of profit than 67% is uncertain. For example, high-turnover customers may purchase higher- or lower-margin goods from the firm’s offerings.^7^

The more general argument against focusing just on the top-20% is that it is but one point on the Lorenz curve. A Lorenz curve is constructed after first rank-ordering individuals, for example according to the amount gambled over the period. The horizontal axis of the diagram on which it is plotted would show the cumulative proportion of gamblers and the vertical axis might show the cumulative proportion of operator income from those gamblers. In many applications it is conventional to show the rank-ordering from lowest to highest but, in the present context, we follow Schmittlein et al. (1993) by craving indulgence for thinking about an ‘inverted’ Lorenz curve where the rank-ordering is from highest to lowest. If the traditional Lorenz curve is inverted, the Pareto ratio may then be read off from the vertical axis at the point on the curve corresponding to 20% on the horizontal axis.^8^ The value of the Gini coefficient will be unchanged whether or not the Lorenz curve is inverted.

Perhaps the Pareto ratio provides a convenient way of comparing concentration across, for example, different gambling products. However, it may not be the most interesting point on an (inverted) Lorenz curve and comparisons of this single point across products may be misleading. For example, in the case of some gambling products, but not others, the player at the 20th centile may have a rather modest level of activity because truly ‘heavy players’ are represented only at points further left on the inverted Lorenz curve.

In advocating use of the Gini coefficient, Fiedler et al. (2019) go to the other extreme. Instead of focusing on a single point on the Lorenz curve, they choose a single statistic which is intended to summarise the shape of the whole curve. As noted above the Gini coefficient is a measure of the deviation of the Lorenz curve from a 45-degree line given by $x=y$. If every customer of a firm in a conventional industry behaves identically, the value of Gini is 0 because the Lorenz curve exactly follows this 45-degree line. The higher the value of Gini, the greater the degree of inequality. Its maximum value is conventionally given as 1 (all the activity is from a single individual).

However, gambling is not a conventional product. In gambling, a typical customer loses money and the loss can be viewed as the amount paid for the entertainment (Eadington, 1999). But, in contrast to other forms of entertainment, some customer visits end with a monetary gain for the consumer rather than the firm. This results in some negative numbers in the data for spending on each account over an observation period. Just as the Pareto ratio may exceed 100% if gamblers are ordered by spending, so also will the Gini coefficient exceed 1 because of the presence of winners in the data. Fiedler et al. (2019) and Wardle et al. (2023) appear to avoid this issue because of the absence of negative values in their data. This may be presumed to be primarily because the questions asked in their surveys elicited responses that recalled amount wagered rather than amount lost. Hence their reported estimates for Gini fall within the traditional range but they do not capture the distribution of spending across players, only turnover.

Fiedler et al. (2019) advocated that gambling operators should be required to include the value of the Gini coefficient as a metric in their regulatory returns. Regulators, they argued, could compare Gini values across operators and products and impose tougher controls where the value of Gini was higher. The rationale was that, where there was higher concentration, a relatively large share of activity would be by heavy players who will often be experiencing harm. Protecting them could be given high priority because any loss of welfare to light gamblers fully in control of their behaviour would be limited (given that they choose to consume only at a low level).^9^

The proposal should be treated with caution. Lorenz curves may cross, which is to say that Lorenz curves with very different shapes can yield the same value for the Gini coefficient. This implies that unambiguous ranking of gambling products, or of the dependence of operators on heavy players at different time points, may not be possible using Gini coefficients on their own. Further, the Gini coefficient is not dependent on the level of what is being measured (e.g. gambling spend), which is an unappealing property in our context. To take a stark example, suppose a gambling operator sells a gambling product on which 1,000 customers each spend £2 per week. Because they all spend the same amount, there is perfect equality and Gini equals 0. But Gini would also equal 0 for a product where 1,000 customers were each spending £200 per week. Here the Gini coefficient fails to distinguish between the two products whereas it is only the second that would be likely to raise questions of whether customers might be harmed by unaffordable expenditure. Again, although it is likely to be associated with harm to some of the players, the second product is made to appear benign because spending displays perfect equality across consumers. Few regulators would be likely to regard a product on which buyers spent £200 per week as one which should be treated with only the lightest of touches.

### Length of Observation Period

Whether choosing to report the Pareto ratio or the Gini coefficient, prior studies have used data sets which cover very different durations from each other, as short as three months in the case of Wardle et al. (2023). This is in itself a source of non-comparability between results across studies because either measure of concentration may be sensitive to duration. Fiedler et al. (2019) argued for a one-year time window to smooth out distortions associated with seasonality in demand. But, even in the absence of seasonality, there is a case for measurement over a relatively long period. Schmittlein et al. (1993) showed by illustration that, in the general case, the Pareto ratio increases with the time-window because, over a short duration such as a month, the calculation does not distinguish between similar levels of spending by infrequent purchasers who may not buy again for several months and heavily engaged buyers who can be relied upon to be present in every future month in the year. This argument would apply similarly to the Gini coefficient.

Deng et al. (2021) tested the sensitivity of a concentration measure to duration in the specific context of gambling. When calculated for individual months within the one-year observation period, the Pareto ratio for their online casino was always close to 80% whereas it was 91.8% when measured using the full period. They present results of calculations for intermediate durations and note that the estimate stabilises as one-year is approached.

### Our Choices

We determined our methodological choices based on reflection from lessons to be drawn from the discussion of prior literature. Confidence in the results to be obtained was strengthened by the opportunity of using account rather than survey data. Further, the large sample size and its spread across a group of operators that commanded a majority of the market in Great Britain were factors which mitigated the risk that the results would not adequately represent the situation facing typical large operators in regulated online gambling.

**Fig. 1:**
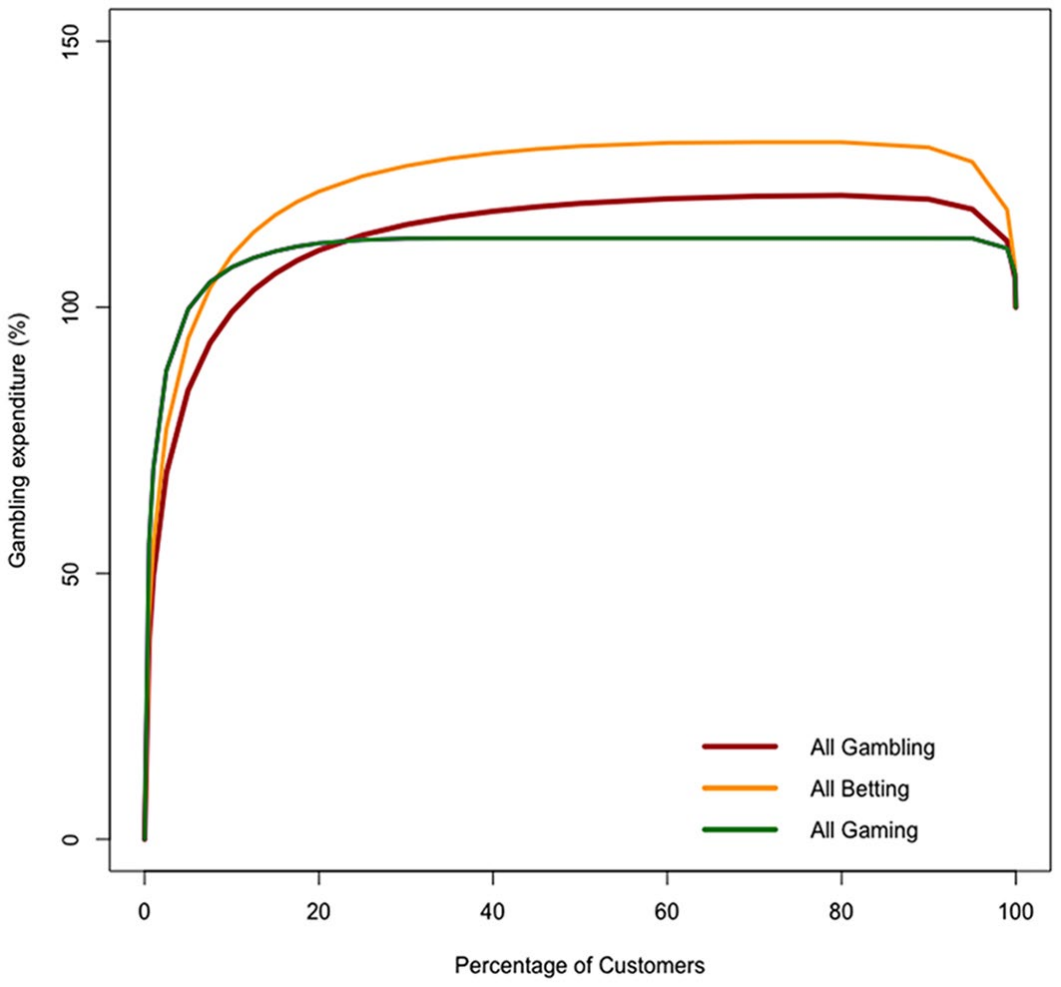
(Inverted) Lorenz curve with accounts ordered by net spending (from highest to lowest)

The choice of what to measure has two elements. A Lorenz curve, from which both the Pareto ratio and the Gini coefficient are derived, shows the percentage of aggregate outcome, *y*, derived from any given percentage of customers, *x* (where *y* and *x* are restricted to the range 0-100). In the literature, outcome is variously considered as spending, turnover or number of gambles. But our interest is to evaluate how financially dependent the industry is on a small group of customers and therefore spending is necessarily our focus outcome. However, we have identified the problem that, if values of *x* also related to customer spending, the Pareto ratio and the Gini coefficient could (and would in our data) be driven much higher than their conventional upper-bounds of 100% and 1 respectively, and we reasoned above that such estimates would in any case be misleading. Indeed, Fig. 1 shows the (inverted) Lorenz curve for ‘all gambling’ (and curves for betting and gaming separately) based on ordering customers in our data set (described in the following section) according to expenditure. On the left of the plot, the highest spenders (i.e. those who lost the most over the period) contribute much more than 100% to the revenue of the industry because their losses have to pay for the wins (negative consumer spending) accrued by the ~ 10% of customers, as ordered by expenditure, who actually win money. The Lorenz curves (inverted in this case) fail to follow the conventional shape, first increasing but turning down towards the right of the diagram where the negative contribution of the winning bettors has its impact.

That the contribution of the highest spending individuals is greater than 100% is somewhat nonintuitive and fails to capture the degree of dependence on the heaviest consumers. Therefore *x* in our report of results will be defined by turnover, i.e. the total amount staked (rather than spent) over the observation period.

Our choice, then, is to express lead findings in terms such as the top-*x*% of customers ordered by turnover (stakes), generate y% of the spending on the product (or, equivalently, the net revenue of the firm). We will report, for online gambling in aggregate and for particular product groups, the result where *x* is set to 20%, i.e. the Pareto ratio. But this alone would be inadequate for comparison between product groups because often the group of players who could reasonably be regarded as the few vital to the viability of the business is much smaller. Thus we will also report values of specific points to the left of the Pareto point on (inverted) Lorenz curves.

For comparison with other studies, we will note values of Gini coefficients but only in passing, because of issues raised above but in general because they relate to the shape of the whole Lorenz curve whereas the research question is about dependence of the industry on the population of customers lying towards its extremity. The Gini coefficients to be reported were calculated to show the degree of inequality in spending across customers rank-ordered by turnover (total stakes). All our measures relate to data from a one-year period, consistent with the recommendation in Deng et al. (2021).

## The Data Set

Each of seven leading operators provided a list of the registration numbers of all accounts held by customers living in Great Britain and used at least once to gamble with the account-holder’s own money (i.e. promotional free bets were excluded) during the one year period running up to June 30, 2019, together with the number of days on which gambling had taken place and the game types (betting, gaming) in which the player had participated over the period. The researchers then used stratified random sampling to select the accounts for which the operators were to supply records. Stratified random sampling was employed in order to capture adequate numbers of sub-groups. For example, a large number of accounts had been used only once whereas there were relatively few very frequent players. Therefore very frequent players were intentionally over-sampled. Thus, of the 20,000 accounts to be requested from each operator 1,000 (5%) were to be of players who gambled only once but 7,000 (35%) were to be of players who gambled on at least 100 days. Weights, equal to the reciprocal of the *ex ante* probability of being included in the sample, were applied (where weights also took into account that the number of records to be requested from each operator was to be the same whereas the seven businesses had very different numbers of account-holders).

Estimates in the present paper are from making projections from the weighted sample. The target sample size was 140,000 but some accounts supplied by the operators proved not to meet the inclusion criteria (e.g. the account address was not in Great Britain or the account had been used during the year only to withdraw funds and not to make a gamble). Deleting these reduced the achieved sample size to 139,152, where these accounts ‘represented’ a total of 10.23 m accounts in the sampling frame. A condition of using the data was that the full sample had to be used in all analysis (i.e. it was not permitted to conduct separate analyses of the businesses of individual operators). Together, from information supplied to us by the Gambling Commission and based on regulatory returns, the seven operators cooperating in the study accounted for 85.5% of the gross gambling yield (total staked minus total pay-outs to winning gambles, i.e. net spending) of all online betting in the regulated sector in Great Britain over the twelve months and 37.5% of the online gaming market (defined as slots, live and virtual casino games, and bingo). The difference reflects both a more fragmented gaming market and that, while all seven operators offered all products, several were brands best known for betting.

Information in the data files was organised differently for betting and gaming activities. For betting, each individual wager was listed including the amount staked and, for cases where the bettor won, the amount credited back to the account after its resolution. In the case of gaming, where, for example, very rapid slots play could involve dozens of individual gambles per minute, such high granularity was impractical and, instead, operators provided summaries of account activity for consecutive 15-minute periods over the whole twelve months. But, as with betting, the information was sufficient to permit us to measure the amount gambled over the year and the amount won or lost by the account-holder. This is the information we use in the present paper.

## Results

All operators contributing data to the study were large businesses providing a ‘full service’ in the sense that they offered the full range of betting and gaming products. Similar to indications from the *Health Survey for England, 2018* (National Health Service, 2019), for which the field work coincided with our data period, participation in betting was much more common than use of gaming products. Estimates from our sample are that 60.8% of accounts with the seven operators in the study were used only for betting, 14% only for gaming and 25.1% for both. But evidently gaming was the more lucrative sector for the businesses. Mean spending per customer for the three groups of accounts were £134.98, £296.20 and £601.91 respectively. And, although only about one-quarter of customers engaged in both betting and gaming, they collectively generated 55% of net spending (i.e. 55% of net revenue for the seven businesses).

**Fig. 2:**
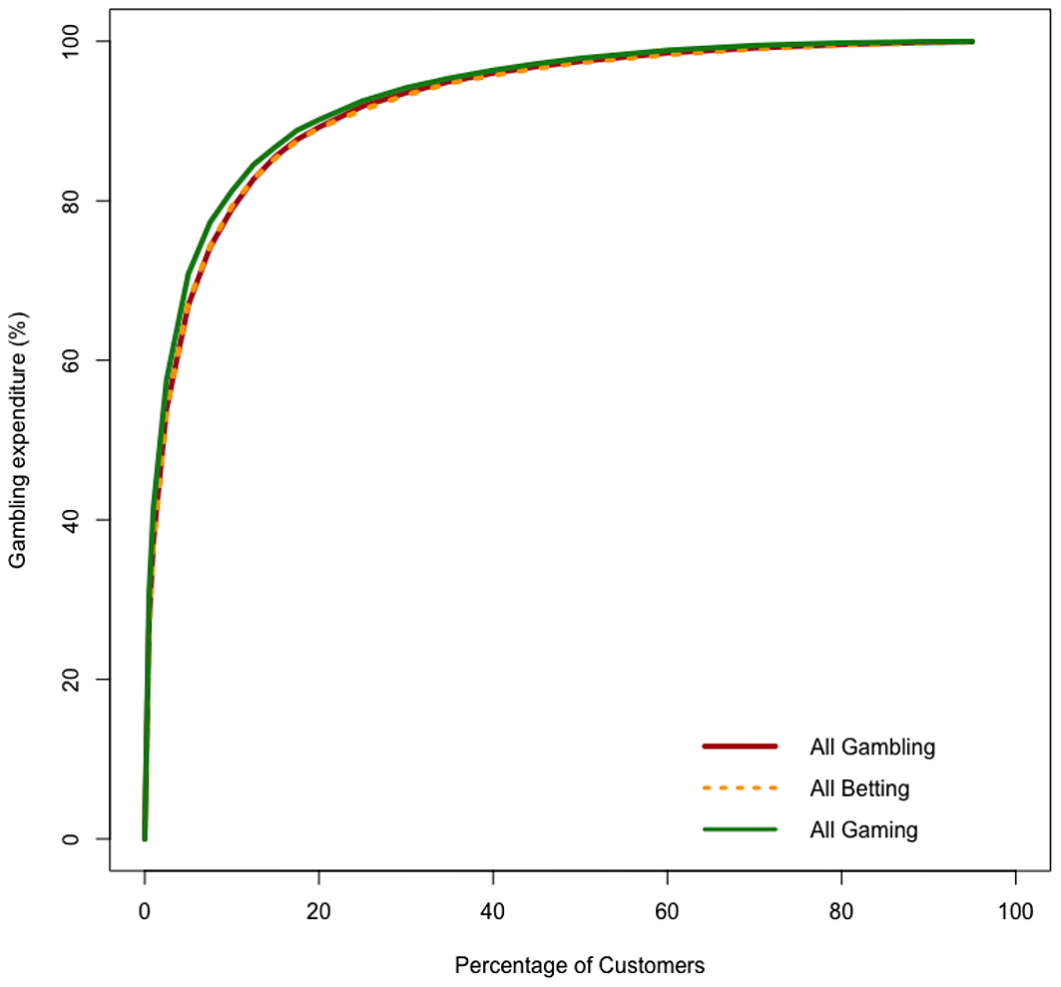
Expenditure (Inverted) Lorenz curves based on ordering customers by turnover (from highest to lowest)

Figure 2 presents the (inverted) Lorenz curves for the aggregate of all gambling activities. The curves for the betting and gaming groups of products considered separately are included for comparison. Each curve represents the full cumulative distribution of operator net revenue from players ordered by amount gambled over the one-year period.^10^ Table 1 highlights three points on these (inverted) Lorenz curves, corresponding to the shares of net revenue extracted from the top-1%, top-5% and top-20% of customers ordered by total amount staked. The table also shows these points for selected individual products within the betting and gaming offerings.

**Table 1:** Selected points on the (inverted) Lorenz curves^a^

	top-1%			top-5%			top-20%			
	% of turnover	% of operator win	stakes threshold (£)	% of turnover	% of operator win	stakes threshold (£)	% of turnover	% of operator win	stakes threshold (£)	
all gambling (*n* = 139,152)	57.67	37.43	70,175.74	82.37	66.94	12,420.00	96.17	89.23	1,388.63	
betting (*n* = 110,203)	52.53	36.42	30,493.45	78.86	66.72	5,635.69	94.91	88.97	710.04	
gaming (*n* = 84,572)	57.14	41.64	116,568.35	82.99	70.82	20,654.90	96.88	90.16	2,019.88	
*selected individual products*										
sports betting (*n* = 101,809)	56.91	34.89	19,628.77	79.75	64.81	3,396.15	94.46	87.83	512.37	
horse/dog betting (*n* = 87,668)	58.55	52.26	20,060.00	83.87	77.68	3,115.20	96.31	91.04	293.41	
bingo (*n* = 16,652)	46.07	34.22	3,398.79	72.65	61.42	581.08	89.59	82.78	102.90	
live casino (*n* = 32,144)	69.34	43.71	72,865.07	89.69	73.78	9,400.00	98.49	91.58	697.00	
virtual casino (*n* = 44,697)	75.23	59.78	65,743.40	92.39	81.71	6,822.00	98.60	93.67	559.00	
slots (*n* = 66,220)	49.75	40.74	89,408.31	79.11	69.94	17,341.70	96.26	90.38	1,740.86	

^a^ The top-x% of users of each product group are defined by turnover (total stakes over one year). Operator win is total amount staked minus total returned in pay-outs on winning gambles. ‘Threshold’ is the minimum turnover required for a customer to qualify for inclusion in the top-x%

In all three curves displayed in Fig. 2, the very steep slope to the left of the diagram shows high concentration of spending by customers/ revenue to the firms. For ‘all gambling’, the Pareto ratio is 89.2%, which is to say that the top-20% of account-holders were responsible for generating 89.2% of the net revenue in these businesses. By contrast, the curves quickly flatten out, indicating that the bulk of customers were very marginal in terms of contribution to the businesses. The bottom-50% delivered just 0.51% of net revenue. These gamblers belong to what Tom et al. (2014) termed the ‘trivial many’.^11^

The estimate of the Pareto ratio supports the notion that online gambling is more dependent on the highest volume customers than may be the case in some other industries. On the other hand, it may not be wholly appropriate to define ‘high volume’ by reference to an arbitrary point on the (inverted) Lorenz curve. The threshold for entering the top-20% of customers could be regarded as somewhat modest, an amount gambled of £1,388.63 over a year, equivalent to an average of £115 per month put at risk. Across all betting, the proportion of stakes retained by the operator rather than returned as winnings was 8.7% and across all gaming activities it was 4.2%. Therefore, even without taking into account that, as evident from Table 1, heavier players tend to achieve less negative rates-of-return, a proportion of customers classified as being in the 20% of ‘vital few’ will have had a net loss only in the region of £100 in the year. Some of these may have been experiencing gambling harm because they were losing heavily on other websites or at land venues. This we cannot observe. But, from the perspective of the businesses analysed here, it could be argued that their customers around the 20th centile could not fairly be described as heavy spenders even if they had high volume relative to the average. Those who exceeded the threshold for entering the top-20% by even two-fold would still often have been spending at or below the limit where, on the basis of evidence from Australia (Dowling et al., 2021), risk of harm is thought to start to rise significantly. Therefore it might be more relevant to focus not on the Pareto point but on points further to the left on the (inverted) Lorenz curve, where spending, even at the one website at which each individual is observed, is beyond safe gambling limits.^12^

Across gambling in the aggregate, the top-10% of customers provided 79.0% of operator revenue and the top-5% just over two-thirds. The top-1% generated 37.4%.^13^ The thresholds for inclusion in these groups were total stakes of £4,568.30, £12,420.00 and £70,175.54 respectively. Given the proportions of turnover retained by operators, a typical customer loss over a year would have been in the low hundreds in the first case, the mid-hundreds in the second case and in the few thousands of pounds in the third case. For context, median household disposable income in the United Kingdom in 2018-19 was £29,400 (Office for National Statistics, 2019).

Gamblers in the top-10% will typically^14^ have been spending above the safer gambling limit proposed by Dowling et al. (2021) though it might be noted that these authors find that only a minority (7–12%) of gamblers exceeding limits experience harm. Within the 10% group, there are smaller groups, such as the top-1%, where typical spending would appear to be at levels which would be challenging to sustain for many British households and where the risk that the customer is experiencing harm would be substantially higher than average. Dependence for 37.4% of revenue on 1% of account-holders is therefore a risk to the operators because the social legitimacy of allowing engagement to the level of the top-1% will inevitably be challenged. All members of the top-1% gambled with more than £70,000 over twelve months.

In some of the literature reviewed in Sect. 2 above, there was a tendency to wish to compare product groups within gambling according to risk of harm by comparing the degree to which each exhibited high concentration of revenue. However, without reference to the absolute levels of activity at relevant points on the distribution, this is likely to be too simplistic an approach. Consider the simplest break-down of the businesses, into betting and gaming. For these sectors, the Pareto ratios were 88.97% and 90.16% respectively. We calculated the Gini coefficients as 0.873 and 0.882.^15^ Using either measure of concentration, the ordering suggests that gaming is ‘riskier’ but the numerical estimates are in fact very similar as between the two product groups. The marginal difference fails to reflect that the absolute levels of volume of gambling are very different across almost the full range of the two (inverted) Lorenz curves. At the 20th centile, the Pareto point, amount wagered is only £710.04 in the case of betting but £2,019.88 in the case of gaming. Notwithstanding that expected loss per pound gambled is a little lower in gaming than in betting, corresponding losses for the typical customer at the 20th centile would be significantly higher.

On the other hand, typical losses at the 20th percentile would still be rather modest in either case. Points further to the left on the (inverted) Lorenz curve involve play at a level which is more obviously concerning. The threshold to enter the top-1% of betting customers is more than £30,000 gambled, and more than £116,000 in the case of gaming. Respectively, these customers generated 36.42% and 41.64% of operator revenue. These are stark figures which illustrate that, even if introduced at unexpectedly high levels, regulatory constraints on account turnover or loss which were applied effectively would make these online businesses non-viable at their current scale of activity.

From the data displayed in Table 1, sports betting and, especially, bingo appear to be the products least compromised by heavy dependence on a small fraction of participants. Even here, however, there are a ‘vital few’ heavy players who account for a significant proportion of spending. In bingo, the top-0.5% of players all staked upwards of £6,737 over the twelve months and collectively generated 26.5% of online bingo revenue at the seven operators. Any limits on, for example, deposits, would threaten a significant proportion of bingo revenue if set at the relatively low levels observed in some jurisdictions.

In the case of horse race betting, any limits introduced would also impinge on the sport itself to the extent that betting operators must pay a statutory levy of approximately 10% of their net revenue from bets on British racing. Receipts from this hypothecated tax are received by the Horserace Betting Levy Board, which distributes it across the sport, principally to support prize money. This funding stream appears to be vital to maintaining the size of the fixture list. According to our estimates, the top-1% of horse race bettors (by amount staked) at these seven (dominant) operators comprised only some 60,000 individuals but their high level of mean loss over the year (£4,199.28) meant that they generated more than half (51.93%) of operator net revenue from horseracing. The share of this used to support racing therefore underpinned much of the sport. The qualification for membership of this top-1% group was an amount staked of £18,585.26. Any restrictions on account deposits or losses would be likely to be at a level which, if applied effectively, would cut off a significant part of the income flowing to racing. Irrespective of the risk to the sport from regulatory intervention to mitigate gambling harm, the dependence on such a small absolute number of individual bettors for more than half of Levy income could in itself be regarded as a risk to horseracing.

## Discussion

Our findings describe the degree of dependence on high-volume customers of large, regulated online gambling businesses operating in a more mature market than studied in pioneering work such as that by LaBrie et al. (2007, 2008). We have also measured concentration for a wider range of online gambling products than prior papers. Our choices on the construction of appropriate measures have differed somewhat from most predecessor work in the field. But, all that notwithstanding, the headline remains the same: almost regardless of product, gambling companies extract a large proportion of their revenue from a small proportion of customers who tend to be gambling to levels associated with elevated risk of gambling harm.

While this headline has remained constant throughout the evolution of research in the field, the threat it poses for the industry has become more urgent. There is growing advocacy for severe restraints to be placed on gambling activity. For example, a Delphi panel of ‘experts’ (Regan et al., 2022) endorsed a radical set of 40 policies intended to curb gambling. Change on the ground is already evident. In mainland European jurisdictions cited in Sect. 1 above, various maximum limits for those using online gambling accounts have been introduced. In Great Britain, the Gambling Commission has implemented rules to deter operators from granting VIP status as an incentive to their heaviest customers and, in its White Paper on gambling reform, published in April, 2023, the Government proposed that ‘detailed’ affordability checks should be carried out on those losing £1,000 in a day or £2,000 over three months, with details to be finalised after a consultation period (Department for Culture, Media and Sports, 2023). All such measures have the potential to be financially very damaging to an industry with the seemingly very high concentration of revenue demonstrated again in our research.

Independent of regulatory threats, any business with very high dependence on a small number of buyers may seek to mitigate the obvious commercial risk. But the options available to gambling companies are more limited than in most industries. Marketing more vigorously to individuals who consume at a low level or currently not at all would be a potential strategy to follow in a non-stigmatised industry but in gambling would be likely to attract the ire of the regulator even if it resulted in lower indices of concentration of revenue. The homogenous nature of many gambling products also limits the applicability of a strategy open to the alcohol industry, which presents a similar picture to gambling (Sheron & Gilmore, 2016), that of persuading moderate drinkers to switch increasingly to premium, higher-margin brands, allowing replacement of revenue from current heavy drinkers (Bhattacharya et al., 2018). Online gambling companies compete with each other in terms of, for example, ancillary content (e.g. sports news) on their websites and mobile ’phone apps, but a bet is unambiguously defined by the odds on an event and so additional consumption expenditure comes from higher stakes rather than a shift to higher quality of the core product.

Eadington (1999) described problem gambling as the Achilles Heal of commercial gambling. Given the greater emphasis on safer gambling in current times, it is likely to prove so for the regulated online gambling sector in the coming years. To date, rapid expansion has been facilitated by channel shift away from offline gambling but this source of growth has its natural limits and is likely to be offset by operators having to forego a significant proportion of revenue either because of the imposition of regulatory limits on spending by individuals or by themselves, under pressure, becoming more proactive in investigating whether their heaviest customers can afford the losses they are sustaining.

In Great Britain, there are signs that, subsequent to the period of our analysis, the largest online operators did indeed take steps to reduce their dependence on high revenue customers. This was acknowledged by the Gambling Commission (2023) which, since 2020, has collected monthly data, with new indices of customer behaviour, from the largest online operators. It has drawn attention to a fall of 8% in the number of customers losing more than £500 per month. In the case of slots games, the product generating the highest revenue for the industry, the proportion of players staking more than £50 per spin had been nearly halved at some operators. There have been significant falls in the proportion of revenue derived from ‘higher spending’ customers. Indeed, it was claimed in Gambling Commission (2023) that one major operator had reduced its proportion of income from ‘high spending customers’ from 19 to 5% over the preceding three years (though no information was given on how high spending was defined). The largest businesses have also declared in the public record that future growth depends on increases in sales to ‘recreational players’ (a term commonly used to refer to lighter users).^16^ From the extremely low contribution to revenue of light users demonstrated in Fig. 1, it seems unlikely that revenue from this group can replace that foregone from the heaviest users to any meaningful extent. This is confirmed by the Gambling Commission data: despite an increase in the number of bets and the number of active accounts, the operators were reporting a fall in gross gambling yield of about 16%, the result of “a policy of withdrawing from what they [the large operators] regard as higher risk staking and losses” (Gambling Commission, 2023).

Perhaps adequate replacement of revenue from the heaviest players could be achieved in the future by shifting a large number of customers to levels of spending currently associated with the margin of the ‘vital few’ group but it is not clear how this could be achieved and would invoke criticism because these spending levels might still challenge safer gambling limits. Perhaps, then, the sustainability of current profit levels at the large British operators may have to rest on their ability to claim significant market share in emerging legal markets, including in American states which have recently authorised online gambling for the first time, replacing lost domestic revenue with enhanced export income.

Voluntarily shedding high-volume domestic customers, if indeed it has occurred, may appear a surprising strategic shift by the British gambling operators. But it could be a response to two considerations.

First, the Gambling Commission’s “approach to enforcement changed significantly in 2017 when it unveiled a new strategy to tackle operators which breach their licence conditions and relevant codes of practice” (Department for Culture, Media and Sport, 2023, p. 115), resulting in much more frequent and higher penalties for non-compliance. Between 2017 and March 2023, penalties totalling £188mn. were imposed on operators, typically for failure to fulfil existing social responsibility requirements to assess the gambling of customers losing large amounts. Potentially, the online context for gambling offers the prospect of operators being able to employ algorithms which track play and, reasonably reliably, identify customers with a high probability of experiencing harm (Deng et al., 2019). Operators are then expected to intervene to check on affordability and then, where appropriate, to help or enforce that the customer scales back their gambling. Repeated public exposure of failure to act accordingly lead to loss of public trust and raise suspicions that lack of action is related to the effect on profits were high volume customers to be constrained. Greater compliance may restore public trust to an extent and reduce the risk of fines.

Second, during 2020–2022, the U.K. Government was conducting a review of gambling legislation and regulation which was widely expected to lead to more prescriptive rules requiring operators to assess whether high spending customers were likely to be experiencing harm. In the event, the White Paper which followed (Department for Culture, Media and Sports, 2023) proposed mandating detailed affordability checks for customers exceeding specified loss thresholds. But the details remained to be worked out during a public consultation. For the industry, these details will be important in terms of how intrusive the checks might be for customers, how ‘unaffordable’ is to be defined, and what action operators would be expected to take where a customer was deemed to be incurring unaffordable losses (for example, will they have to exclude the customer altogether? ). From our measures of the concentration of revenue, a rigorous interpretation of affordability checks would be expected to eliminate a high proportion of the income of the regulated industry. One way of meeting this threat may have been to seek to demonstrate in advance a willingness to comply with current regulatory requirements and to proactively address gambling harm among high value clients. On investigation, many of these will prove to be ‘healthy and wealthy’ high rollers (Deng et al., 2021) whose gambling is under control and whose high spending reflects either high income or very strong preference for gambling as a leisure good. Such customers could be permitted to continue to deliver income to the industry. If it can persuade politicians, regulators and public opinion that it can be trusted to comply, the industry could therefore retain at least part of the ‘vital few’ group whereas a strong definition of ‘unaffordable’ or very intrusive financial checks which customers might refuse to accept could threaten losing nearly it all. That such persuasion by the online industry may prove to be difficult in the light of past failings underlines that it has paid too little attention to long-run sustainability and the maintenance of its social licence to operate. These failings are likely to have been evident in other gambling jurisdictions beyond Great Britain.

## Data Availability

The data analysed in this paper consist of some 140,000 records of individual accounts held across seven different operators licensed by the Gambling Commission in Great Britain. For each account, the record, covering a one-year period, included all transactions, customer use of safer gambling tools, and safer gambling contacts made by the operator. The operators jointly imposed a condition that the data provided to the Project Manager selected by the funder could not be shared beyond the researchers it employed to conduct the analysis. The data are therefore not publicly available.

## Footnotes

1 ‘Great Britain’ refers to England, Scotland and Wales. The other constituent nation of the United Kingdom, Northern Ireland, has its own framework of gambling law and regulation. Thus all references in the paper to data and policy in Great Britain apply only to Great Britain.

2 For comparison with other leisure services, consumer spending on attending sports events and cinemas in 2022 in Britain are estimated as £1.5bn. in each case (Mintel, 2022a, 2022b). Video game purchases in Britain were estimated at £4.3bn. in 2021 (ERA, 2021).

3 The figures here exclude gambling with the National Lottery.

4 The Gini coefficient, explained in greater detail in the following sub-section, is an index of concentration which can take values between 0 and 1, where a higher number implies greater inequality. Here, if Gini = 0, every gambler would contribute the same amount to aggregate gambling. As the value of Gini increases, this reflects increasing domination of total gambling by heavier players.

5 Wardle et al. also calculated Gini coefficients for individual products such as casino games using data only from regular race and sports bettors. These estimates seem unlikely to show accurately the dependence of revenue from those products on high spenders. For example, there will be many customers who play casino games but are not regular sports bettors and there is no reason to suppose that their pattern of engagement is similar to that of those who also participate in race and sports betting.

6 In sports betting, the discontinuity in the distribution was observed at the 99th centile (LaBrie et al., 2007).

7 Using a much smaller sample, McCarthy and Winer (2019) were able to estimate the Pareto ratio for the case where the top-20% of customers were still defined by turnover but their contribution to the firm measured by profit. This raised the Pareto ratio by 30% points. The variation in the value of the Pareto ratio reveals the sensitivity of estimates to different choices as to what is being measured. This makes comparison of results between different studies of gambling difficult.

8 Schmittlein et al. (1993) argue for presenting an inverted Lorenz curve in the context of marketing because marketing managers focus on the top-20% of customers. Given that business risks in gambling outlined in Sect. 1 all relate to dependence on the heaviest players, we choose to follow this precedent.

9 Whilst the argument has some appeal, it should be pointed out also that many of the heaviest players will not be experiencing problems. The consumption benefit those players lose from the imposition of restrictions should also be taken into account in any policy impact exercise.

10 The curves were constructed from data points corresponding to centiles on the x-axis. Were the plot to show the effect of adding each individual customer one at a time, the curve would show a small downward segment each time when the new bettor had been a winner over the year.

11 Of course, whilst half of all customers transacting during the year made a trivial contribution to the businesses during that year, they may still be worth retaining for the longer run because a small proportion of them may transition in the future to membership of the ‘vital few’.

12 Dowling et al. (2021) suggested a limit of AUD380-615 per year depending on which of two state-level data sets they used. At the average exchange rate over 2021, this would convert to approximately £207–335.

13 In analysing very large-scale transactional data set for spending on mobile video games, Zendle et al. (2023) identified a set of games where concentration was highest. They termed these ‘hyper-Pareto’ games and reported that, on average, the top-1% of consumers accounted for 38% of revenue from them. This set of video games included casino- and card-like games but also games in some other genres.

14 Some will have not spent anything over the year as they finished net winners. However, the data reveal many more net losers than net winners.

15 For the aggregate of gambling, Gini was 0.875.

16 See, for example, Flutter Entertainment plc (2022, p. 18).
